# Testing a potential alternative to traditional identification procedures: Reaction time-based concealed information test does not work for lineups with cooperative witnesses

**DOI:** 10.1007/s00426-017-0948-5

**Published:** 2017-11-27

**Authors:** Melanie Sauerland, Andrea C. F. Wolfs, Samantha Crans, Bruno Verschuere

**Affiliations:** 1 Section Forensic Psychology, Department of Clinical Psychological Science, Faculty of Psychology and Neuroscience, Maastricht University, P.O. Box 616, 6200 MD Maastricht, The Netherlands; 2 Department of Clinical Psychology, University of Amsterdam, Amsterdam, The Netherlands

## Abstract

Direct eyewitness identification is widely used, but prone to error. We tested the validity of indirect eyewitness identification decisions using the reaction time-based concealed information test (CIT) for assessing cooperative eyewitnesses’ face memory as an alternative to traditional lineup procedures. In a series of five experiments, a total of 401 mock eyewitnesses watched one of 11 different stimulus events that depicted a breach of law. Eyewitness identifications in the CIT were derived from longer reaction times as compared to well-matched foil faces not encountered before. Across the five experiments, the weighted mean effect size *d* was 0.14 (95% CI 0.08–0.19). The reaction time-based CIT seems unsuited for testing cooperative eyewitnesses’ memory for faces. The careful matching of the faces required for a fair lineup or the lack of intent to deceive may have hampered the diagnosticity of the reaction time-based CIT.

## Introduction

Eyewitnesses’ memory for the face of a perpetrator is commonly tested by means of an identification procedure, for example, a lineup or showup. It is well established that eyewitnesses who are submitted to such a procedure can help solving a crime by pointing out the actual perpetrator, but it is equally well known that eyewitnesses can err. In the worst case, a wrongful identification decision can lead to a wrongful conviction while allowing the guilty party to remain free and reoffend. Wrongful identifications were involved in 70% of the wrongful convictions uncovered by the innocence project (innocenceproject.org; cf. Kassin et al., 2012; Wells et al., 1998). While identification accuracy can vary widely across conditions, different meta-analyses show that, on average, accuracy for six-person lineups (i.e., seven answer options: all six lineup members and the option to reject the lineup) revolves around 50% (e.g., Clark et al., 2008; Fitzgerald & Price, 2015; Steblay et al., 2011). Although proper lineup construction and administration can increase accuracy rates (e.g., Brewer & Palmer, 2010), the risk of false identifications remains and continues to be a major concern in the field. Scholars have recently questioned researchers’ sustained confinement to the traditional eyewitness identification paradigm (Brewer & Wells, 2011; Wells et al., 2006). More specifically, it has been argued that existing research may not be radical enough, with new procedures merely constituting adaptations of existing ones (Dupuis & Lindsay, 2007), rather than generating fundamentally new approaches for testing eyewitnesses’ memory for faces. Existing procedures rely on explicit identification, often after some deliberation. One possible source of error is the constructive identification through reasoning (i.e., the culprit is likely to be included and number 4 looks most like him, so it must be number 4). More gross errors in explicit identification may come from uncooperative eyewitnesses that deliberately point to the wrong person (e.g., to protect someone else; being bribed; after being threatened; see Leach et al., 2009; Parliament & Yarmey, 2002). In other words, explicit identification is prone to subtle biases in human decision making and strategic misidentification. One alternative might be to rely on indirect measures. Such responses are attractive in the sense that they may be unintentional, uncontrollable, goal independent, autonomous, purely stimulus driven, unconscious, efficient, or fast (Moors & De Houwer, 2006). First evidence supporting the idea that indirect measures can provide information about face recognition comes from two studies with pre-school and school children (Newcombe & Fox, 1994; Stormark 2004). In these studies, participants first viewed a slide show of previously familiar faces (playmates or previous classmates) and unfamiliar faces, while their skin conductance, heart rate, or both were recorded. Subsequently, direct face recognition responses were collected. Although both direct and indirect measures scored above chance in both studies, the indirect measures outperformed direct recognition decisions. In the current line of research, we embraced the call for exploring a potential adaption of the identification procedure in a venture that tested an indirect index of eyewitness identification: the concealed information test (CIT; Lykken 1959).

The CIT is a well-established memory detection technique (for a comprehensive review, see Verschuere et al., 2011). At first, the CIT looks much like a multiple-choice examination, presenting the examinee with the correct answer embedded amongst a series of incorrect answers. The CIT is used when the examinee may not be able or willing to explicitly identify the correct alternative, and, therefore, does not rely upon an explicit answer but rather on more automatic responses to determine recognition. Suppose an exclusive blue Porsche has been stolen, and the police has a suspect that denies any involvement or knowledge about that theft. The suspect of the car theft could be asked about the stolen car: Was it ….a white Bentley?...A green Mercedes?...*A blue Porsche?*...A yellow Ferrari?...A black Jaguar? Stronger (e.g., electrodermal) responding to the actual stolen car compared to the other cars, is taken as an index of recognition. When combining several questions, the CIT can detect concealed recognition with high validity. Reviewing a range of indices, varying from event-related potentials (ERPs) to reaction times, Meijer et al. (2016) reported the diagnostic efficiency of the CIT (i.e., the area under the curve) to be around 0.82–0.94. This means that in such studies, a randomly chosen person with recognition has an 82–94% chance to respond stronger in the CIT than a randomly chosen person without recognition. In recent years, there is growing interest in the use of reaction times as the response measure in the CIT (for a review, see Suchotzki et al., 2017). Response times can be administered and analyzed cost and time efficient, requiring a single computer. In the reaction time-based-CIT, the answer alternatives are presented briefly, one by one, on the computer screen. To assure attention to the stimuli, the examinee engages in a binary classification task, pressing a unique button for a set of stimuli learned just before the test (i.e., the targets) and another button for all other stimuli (including the correct answer or probe, as well as all foils, called irrelevants). Building on the example above, the examinee may be explained that the CIT will examine recognition of the stolen car, and asked to press the YES button whenever encountering the target (a red Maserati) and the NO button for all other cars. For the innocent examinee, all NO-reaction times will be roughly similar. For the guilty examinee, the blue Porsche will stand out and grab attention. Longer reaction times for the blue Porsche as compared to the other NO-reaction times provides an index of recognition. After the initial validation of the reaction time-based CIT (Farwell & Donchin, 1991, Seymour & Kerlin, 2008; Seymour & Schumacher, 2009; Seymour et al., 2000), several recent well-powered studies have confirmed its diagnostic efficiency (Kleinberg & Verschuere, 2015, 2016; Verschuere et al., 2015, 2016; for a discussion of its boundary conditions and limitations, see Verschuere et al., 2011; and Meijer et al., 2016).

Meijer et al. (2007) conducted two studies to examine whether the ERP-based CIT is sensitive for concealed face recognition. In their first experiment, the CIT was capable of picking up recognition of the faces of siblings and close friends. In their second experiment, the CIT did not show students’ recognition of their faculty professor faces. Seymour and Kerlin (2008) had participants memorize a set of previously unknown faces, and the reaction time-based CIT showed high accuracy in concealed face recognition. The stimuli used in these studies, however, were not typical of eyewitness identification, because the correct faces were either very familiar or well memorized rather than incidentally encountered as in the case of eyewitnesses. In addition, they were not matched in terms of their outer appearance. As such, they would not meet requirements of a formal identification procedure in an investigation (cf. Technical Working Group for Eyewitness Evidence 1999; Wells et al., 1998). More specifically, Wells et al.’s (1998) rule 3 concerning the structure of lineups and photospreads of states:

The suspect should not stand out in the lineup or photospread as being different from the distractors based on the eyewitness’s previous description of the culprit or based on other factors that would draw extra attention to the suspect. (p. 630).

This rule is further specified with the fit-description criterion which stresses the importance that distractors should fit the eyewitness’s verbal description of the perpetrator (Technical Working Group for Eyewitness Evidence 1999; Wells et al., 1998). Thus, when the eyewitness describes the perpetrator as ‘young, white female, blond hair’, the lineup should consist of young white females with blond hair.

Lefebvre et al. (2007) were the first to propose the CIT for the purpose of eyewitness identification, namely, to use incidentally encountered faces, and to match faces following guidelines for eyewitness identification. Participants watched four mock crimes across two testing sessions. In the perpetrator-present conditions, participants were presented with the photograph of the perpetrator, the victim, and five foils, one by one, on the computer screen, while electrophysiological recordings were made. Deviating from the classic CIT procedure, participants could respond to each picture by pressing one of three buttons, indicating that this picture depicted the perpetrator, the victim, or another person. In other words, participants made an explicit identification in this ERP-based CIT. The CIT revealed recognition of the perpetrator, and so did explicit identification. While the results point to the potential of the CIT for cooperative eyewitness identification, the electrophysiological index of recognition may have been evoked by the explicit identification. In a second ERP-based CIT study (Lefebvre et al., 2009), the effects were replicated, but also extended by examining the role of active concealment. In the deceptive condition, participants concealed the identity of the perpetrator from the experimenters by pressing the button that corresponded with an innocent individual, rather than perpetrator. Results confirmed the earlier finding, showing that even when trying to conceal their knowledge, the CIT revealed recognition of the perpetrator’s face.

Taken together, there is preliminary evidence that the ERP-based CIT may be useful for testing the facial memory of cooperative eyewitnesses. In the present research line, we examined whether the findings extend to the reaction time-based CIT, which is much easier to apply. This was tested in a series of five experiments. We expected that the recognition of a face previously encountered in a stimulus event (probes) would be reflected in longer reaction times, compared to reaction times for irrelevants.

## Overview of the studies

Participants witnessed a crime involving one or more individuals. The subsequent reaction time-based CIT assessed face recognition of the individuals involved in the crime. Using the classic CIT procedure, participants pressed one specific key for all stimuli (i.e., irrelevants and probes), except for the target stimulus that was memorized prior to the CIT.^1^ The progression of the five conducted experiments can be described as follows: in Experiment 1, one stimulus film depicting four actors who played a thief, a victim, and two bystanders was used. The lineup referring to each actor was presented prior to the referring CIT to receive a lineup performance measure that was unimpaired by CIT presentation. In all subsequent experiments, the CIT was presented first, to obtain CIT performance that was unimpaired by participants’ lineup decision. The use of only one stimulus film in Experiment 1 raised the question whether diverging findings could be attributed to certain roles the film featured (i.e., more attention paid to the thief than a bystander) or characteristics of certain actors (e.g., higher or lower distinctiveness). Therefore, we used different stimulus film versions for all subsequent experiments in which actors switched roles across versions, while the plot was identical.

Following null findings and contradictory results in Experiments 1 and 2, and emerging insights into the validity of the reaction time-based CIT, we realized that we may have used a suboptimal CIT protocol. Indeed, Verschuere et al. (2015) showed that using a separate CIT per probe (i.e., one for victim, one for thief, etc.) reduced accuracy and that it is recommended to use one CIT that presents all items completely intermixed (see also Lukasz et al., 2017). In Experiments 3–5, we, therefore, administered such a multiple-probe CIT, in which all probes, that is, all actors that appeared in the stimulus event and all corresponding irrelevant items were presented in random order. Following small effect sizes in Experiment 3, we considered the possibility that our stimulus films had not allowed for sufficient encoding of the actors’ faces. We, therefore, prepared a less complex stimulus film with only two actors and optimal viewing conditions (long facial viewing time, including close-ups, for both actors) for Experiment 4. Indeed, small but significant effects materialized for the two actors (thief and victim) in this experiment. The final experiment (Experiment 5) additionally addressed three issues: for one, Experiment 5 included an additional practice block and a minimum proportion of accurate reactions during practice before a participant could move on to the actual CIT. Second, a virtual reality event was used instead of a real life film, to be able to better control the actions and exposure of the subjects featured in the mock crime and to offer participants a more realistic experience of the mock crime (cf. Gorini et al., 2007; Kim et al., 2014; Riva 2005; Schultheis & Rizzo, 2001). Finally, we included two control objects in the stimulus event. Finding an effect for the objects but not the faces would replicate earlier findings concerning objects (e.g., Suchotzki et al., 2014; Verschuere et al., 2004; Visu-Petra et al., 2012), showing the validity of the CIT for objects and strengthen the conclusion of the absence of an effect for lineup faces. Anticipating the results, we found a CIT effect for objects, but not lineup faces. Comparison of the methodology in the current studies and CIT research in memory detection in suspects opens new perspectives on when reaction time-based CIT serve as a useful tool to diagnose face recognition in cooperative eyewitnesses.

## Method

Data are publically available using the following link: http://hdl.handle.net/10411/2MUUTT.

### Participants

In total, 436 participants were tested, 35 of whom were excluded. More specifically, these participants did not press the accurate key (i.e., left shift key for targets, right shift key for irrelevants, and probes or vice versa) in 50% of the trials on one response category (i.e., responses to probes, targets, or irrelevants; following Lukasz et al., 2017; Kleinberg & Verschuere, 2015, 2016; Verschuere & Kleinberg, 2016; Verschuere et al., 2015). The numbers of included participants in Experiments 1–5 were 55, 107, 84, 75, and 80, respectively (*N* = 401; 299 women and 102 men, M_age_ 21.44 years, SD 2.48). Participants were mostly Bachelor (88.0%) or Master students (9.7%) who studied at the Faculties of Psychology and Neuroscience (80.9%), Health, Medicine and Life Sciences (6.6%), the School of Business and Economics (4.3%), or other (8.2%). The most common native languages were German (46.5%) and Dutch (31.2%; for Experiments 2–5; native language was not assessed in Experiment 1). Participants received study credit or a gift voucher in return for their participation. The research line was approved by the research board of the faculty.

### Design

A within-subjects factorial design contrasting reaction times to probes vs. irrelevant faces was employed for all experiments.

### Materials

#### Stimulus events

Four different stimulus events were used. They depicted a theft (Experiments 1–4) or the vandalism of a car (Experiment 5) and included one or two perpetrators, a victim, and sometimes one or two bystanders. The number of actors involved in the events was either four (Experiments 1–3) or two (Experiments 4 and 5).

##### Experiment 1

The first stimulus film involved four actors (thief, victim, two bystanders) and depicted the theft of a wallet in a student cafeteria (duration: 5:05 min). A detailed description can be found in Sauerland, Sagana, and Sporer (2012).

##### Experiments 2 and 3

For these studies, four different stimulus film versions depicting the theft of a purse in a bar were used. Across film versions, the four female actors switched the roles of the thief, accomplice, victim, and a bystander, while the plots were identical. This was to avoid possible confounding effects of actor and role. For example, if only one film version is used, it is unclear whether an effect might be attributable to the characteristics of a particular person (i.e., distinctive features) or role (e.g., more attention paid to the thief than a bystander). All versions lasted approximately 3:20 min. A detailed description can be found in Sauerland et al. (2014).

##### Experiment 4

Two film versions depicting the theft of a cell phone, involving a thief and a victim, were created (duration 1:13 min). Analogous to Experiments 2 and 3, the two female actors switched the roles of the thief and victim across film versions. The action can be described as follows: a young woman (i.e., the subsequent thief) rushes from a cafeteria to the train station when she runs into another young woman (i.e., the subsequent victim), resulting in both of their bags falling on the ground. While the thief yells angrily at the victim, both pick up their bags and their contents that had fallen; then they walk away. When the victim searches her phone in her bag, she cannot find it and runs after the thief. The thief is seen running towards the train holding the victim’s phone in her hand.

##### Experiment 5

This experiment used a virtual reality event as stimulus event. This allowed for more control over the actions and exposure of the individuals and objects. In this 1:05 min event, two young women walk through a lighted city street at night. One (woman 1) plays music on her phone, while the other one (woman 2) drops a coke bottle (object 1). Woman 1 walks up to a parked car and jumps on the motor hood to dance. Across the street, the observer sees a building with a neon Casino sign (object 2). Woman 2 dances next to the car and later kicks off one of its side mirrors. When a car drives around the corner, both women run away. The faces of both women can be seen for most of the duration of the film. Each of the two roles could be played by two avatars each, resulting in four identical event versions with the avatar constellations AB, Ab, aB, and ab.

#### Reaction Time-Based Concealed Information Test

In the beginning of the CIT, participants are instructed to press the right shift key as fast as possible in response to a facial stimulus, with one exception, the target. For this stimulus, they should press the left shift key rather than the right one. Participants are then presented with the target for 30 s, accompanied by instructions to encode this face. In a practice block, participants were provided with feedback (good, wrong, or too slow). All CIT stimuli were shown twice and participants were given 1500 ms to react before the next stimulus was shown following an inter-stimulus interval of 1000 ms. The size of the facial stimuli was 260 pixels × approximately 220 pixels. In Experiment 5, two practice blocks (rather than one) were conducted, with an optional third one if participants had more than 50% errors or misses in the second practice block. This served to decrease the number of wrongful responses and subsequent exclusion experienced in the former experiments. Following the practice block, the experimenter left the room and the actual task began. Every stimulus was presented 21 times (Experiments 1 and 2: 20 times) with presentations in random order, resulting in 294 to 588 trials. In Experiments 4 and 5, the question “Do you recognize this?” above every stimulus and the labels “YES” and “NO” on the left and right sides of the screen were added. This was to increase the difficulty of inhibiting a left (YES) response, while the participant actually did recognize the face. In this phase, no feedback was given. The use of the left vs. right shift key was counterbalanced across participants. The methodological specifics of the CITs of each experiment are summarized in Table 1.

**Table 1:** Methodological specifics of five experiments

	Experiment 1	Experiment 2	Experiment 3	Experiment 4	Experiment 5
*N*	55	107	84	75	80
Cover story	Yes	Yes	Yes	No	No
Stimulus event	Staged mock video	Staged mock video	Staged mock video	Staged mock video	Virtual reality event
Duration of event	5:05	3:20	3:20	1:13	1:05
Event versions	1	4	4 (same as Exp 2)	2	4
Number of actors	4	4	4	2	4
Number of roles in stimulus event	4	4	4	2	2
Number of objects included	0	0	0	0	2
Number of practice blocks	4 (1 per role)	4 (1 per role)	1	1	2
CITs	4 (1 per role)	4 (1 per role)	1	1	1
CIT test protocol	1 person	1 person	Multiple persons	Multiple persons	Multiple persons
Number of stimulus presentations (blocks)	20	20	21	21	21
Number of trials	560 (140 per CIT)	560 (140 per CIT)	588	294	588
Lineup presentation	Before each CIT	After each CIT	After CIT	After CIT	After CIT

In Experiments 1 and 2, the CIT stimuli presented included one probe (i.e., the face of one of the persons seen in the stimulus film), a target (the face participants were instructed to encode at the beginning of the CIT), and five irrelevants (i.e., foils). Participants were successively presented with four different CITs, one for each probe. In Experiments 3–5, only one CIT was administered, which included multiple probes, namely, all of the individuals that they had seen in the stimulus film. For Experiment 3, this means that the pictures presented in the CIT included four probes, four targets, and 4 × 5 = 20 irrelevants. The CIT of Experiment 4 included two probes, two targets, and 2 × 5 = 10 irrelevants. The CIT of Experiment 5 included two facial probes and two object probes, two facial targets and two object targets, 2 × 5 = 10 facial irrelevants, and 2 × 5 = 10 object irrelevants.

#### CIT and lineup photos

##### Facial pictures

For taking the facial pictures of probes, targets, and irrelevants, individuals took jewelry, eyeglasses, and hair accessories off and wore their hair loose. The clothing of each person differed from one another and the probes additionally wore different clothing in the film than in the photograph. The photographs were taken against a white wall and edited to display a person from the collarbone up. The selected pictures fit the general description of the actors depicted in the different stimulus events (i.e., the probes). More specifically, for each actor, six matching pictures were selected. One of these pictures was selected to serve as target and the remaining five pictures served as irrelevants in the CIT. In Experiments 1 and 3, one target was pre-selected at random for all participants, whereas in the other experiments, a target was randomly selected for each participant. For the virtual reality event, seven avatars that matched in their general person description were created for each of the two roles (i.e., 14 avatars). Two avatars each were selected to appear in the different stimulus event versions (i.e., four avatars). Analogous to Experiments 1–4, the avatars from the stimulus event served as probes, one avatar each served as target, and the remaining avatars served as irrelevants.

##### Object pictures

Fourteen object pictures were created for Experiment 5. The pictures of a coke bottle and a casino sign were expected to be salient stimuli in the stimulus event (Kleinberg & Verschuere, 2015; Lieblich et al., 1976). Six additional objects falling into the categories consumer goods (hamburger, pack of cigarettes, can of beer, chocolate bar, bag of French fries, and bottle of Whiskey) and façade decoration (Hotel sign, Advent wreath, Dutch flag, Art show sign, carnival garland, and occupation banner reading “This is ours”) were created to serve as targets and irrelevants (foils). The objects that served as targets/irrelevants were randomly selected for each participant.

#### Lineups and lineup construction

The facial pictures described above were used to construct the actor-present and actor-absent lineups.^2^ Lineups were composed of six photographs numbered 1–6 that were arranged in two rows of three pictures (i.e., a simultaneous lineup). All distractors and the replacement (i.e., extra distractor added to actor-absent lineups) fitted the general descriptions of the referring actor, as determined by presenting independent samples of mock witnesses (*n*s between 20 and 31) who had not viewed the stimulus event with a description of each actor together with the referring lineup (e.g., ‘She is about 20 years old. She has long, brown hair. She has a slim to normal figure’). These mock witnesses were then asked to select the person from the lineup who matched the description best (Doob & Kirshenbaum, 1973). Effective lineup sizes for actor-present and actor-absent lineups, determined as Tredoux’s *E*s, were satisfactory and ranged from 3.2 to 5.6 of a possible 6 (M 4.2; Tredoux 1998, 1999).

### Procedure

Participants signed the informed consent form and provided demographic data. Before watching the stimulus event, participants in Experiments 1–3 were instructed to pay close attention to the film, because they would be asked questions about it later on. In Experiments 4 and 5, participants were instructed to pay particular attention to the faces and to encode them as detailed as they could. In Experiment 5, participants were given additional instructions about the use of the virtual reality goggles and handed headphones once they had put on the goggles. They then first saw an orientation environment, which consisted of a big open space, and which allowed them to check if the goggles were placed correctly and gave the participants the chance to get used to being in a virtual reality environment. Then, the CIT task was started. The final part of the experiment was the administration of the lineups; one for each person or avatar that appeared in the stimulus event. Deviating from the described procedure, in Experiment 1, each of the four CITs was preceded by the matching lineup, whereas in Experiment 2, each of the four lineups was preceded by the matching CIT. In Experiment 1, only actor-present lineups were used; in Experiments 2 and 3, the thief and bystander 1 lineup were either both present or both absent, as were the victim and bystander 2 lineup (i.e., two lineups were always absent, and two were present); and in Experiments 4 and 5, actor presence was completely counterbalanced. In Experiment 1, the sequence of the lineups was fixed (Thief-Victim-Bystander 1-Bystander 2); in Experiments 2 and 3, we used a Latin square (Thief-Victim-Bystander 1-Bystander 2 vs. Victim-Bystander 1-Bystander 2-Thief vs. Bystander 1-Bystander 2-Thief-Victim etc.); in Experiment 4, lineup order (thief-victim vs. victim-thief) was counterbalanced; and in Experiment 5, lineup order was random. Testing sessions lasted approximately 30–40 min. The debriefing followed after termination of data collection. A summary of the procedural specifics of the CITs of each experiment can be found in Table 1.

## Results

### CIT data preparation and overview of analyses

Prior to data analyses, those trials with wrongful responses and reaction times faster than 150 ms (i.e., inattentive responding) or slower than 1500 ms were removed from the data set.^3^ Next, data were aggregated to result in the average reaction times per stimulus type per participant and probe (e.g., for Experiment 1, 2 × 4 variables would be computed: the mean reaction times to the probes and irrelevants, referring to the thief, victim, bystander 1, and bystander 2). For each experiment, a paired sample *t* test contrasting probes vs. irrelevants was computed per role. Finally, a weighted mean estimate of the effect size across all five studies was established.

### Comparison of reaction times to probe and irrelevants

Across the five experiments, we conducted 16 tests to compare probe vs. irrelevant reaction times. Eight of the tests showed no effect (|*d*| ≤ 0.20), and five tests displayed small effects into the expected direction; one a moderate and one a large effect into the expected direction. One test showed a small effect in the opposite direction. Table 2 provides the mean reaction times (and SDs) in response to facial probes and irrelevants and the inferential statistics.

**Table 2:** Reaction times, standard deviations, and inferential statistics for the pairwise comparisons of the reaction times for probes and irrelevant stimuli (including reaction times 150–1500 ms)

Study	Role	Played by actor	*df*	*t*	*d*	*p*	Mean response time in ms (SD)	
							Probes	Irrelevants
1	Thief	A	54	− 0.51	− 0.07	.610	421 (76)	424 (69)
	Victim	B	54	3.72	0.50	< .001	466 (84)	444 (64)
	Bystander 1	C	54	0.68	0.05	.499	426 (84)	423 (68)
	Bystander 2	D	54	6.17	0.83	< .001	454 (74)	419 (64)
2	Thief	EFGH	106	− 0.11	− 0.01	.913	374 (70)	374 (58)
	Victim	EFGH	106	− 2.16	− 0.21	.033	369 (64)	376 (58)
	Bystander 1	EFGH	106	− 1.41	− 0.14	.161	367 (61)	371 (53)
	Bystander 2	EFGH	106	− 0.34	− 0.03	.734	370 (63)	371 (56)
3	Thief	EFGH	83	1.70	0.19	.093	494 (76)	485 (55)
	Victim	EFGH	83	2.16	0.24	.034	497 (76)	484 (53)
	Bystander 1	EFGH	83	0.96	0.10	.341	486 (72)	481 (57)
	Bystander 2	EFGH	83	2.09	0.23	.040	497 (81)	484 (54)
4	Thief	IJ	74	2.48	0.29	.015	479 (64)	466 (51)
	Victim	IJ	74	2.12	0.25	.037	479 (61)	469 (55)
5	Woman 1	KL	79	− 0.99	− 0.11	.324	545 (81)	551 (68)
	Woman 2	MN	79	2.23	0.25	.029	535 (77)	521 (62)
All 5 studies	Across roles				0.14 (0.08, 0.19)			
Experiments 2–5	Across roles				0.10 (0.05, 0.16)			

In Experiment 5, two control objects were included in the CIT. Replicating earlier findings, the reaction times for probes were slower (M_O1_ 454 ms, SD_O1_ 58; M_O2_ 451 ms, SD_O2_ 48; M_collapsed_ 453 ms, SD_collapsed_ 47) than for irrelevant stimuli (M_O1_ 442 ms, SD_O1_ 40; M_O2_ 440 ms, SD_O2_ 38; M_collapsed_ 441 ms, SD_collapsed_ 38), *t*(79) = 2.63, *p* = .010, *d* = 0.29 (object 1), *t*(79) = 3.50, *p* = .001, *d* = 0.39 (object 2), *t*(*79*) = 4.26, *p* < .001, *d* = 0.48 (collapsed).

### Meta-analysis across five studies

The five studies together yielded 16 effect size estimates. Using the reciprocal of the sampling variances as weights (cf. Gibbons et al., 1993), a weighted mean estimate of the effect size yielded an average effect size of 0.14 (95% confidence interval: 0.08; 0.19), indicating that across the five studies, a very small effect size materialized. We reran the meta-analysis excluding Experiment 1. This was to account for the fact that in this experiment, the CIT outcome may have been impacted by the preceding lineup task, a procedural detail that may be sufficient to create a deviant response in the subsequent CIT. The yielded average effect size across Experiments 2–5 was 0.10 (95% confidence interval: 0.05; 0.16), a very small effect.

In addition, we reran the meta-analyses including only those participants who correctly identified the actor from an actor-present lineup. The results showed a small average effect size when looking at all five experiments [mean *d* = 0.38 (95% confidence interval: 0.24; 0.52)], whereas there was a very small effect, on average, when Experiment 1 was excluded [mean *d* = 0.15 (95% confidence interval − 0.06; 0.36)].

### Eyewitness identification performance from traditional lineups

Table 3 shows the identification accuracy rates split by experiments and probes. The data concerning Experiments 2–5 must be treated with caution. This is because in these experiments, the lineup task was preceded by the CIT task. This familiarizes participants with the stimuli presented in the subsequent lineup and possibly introduces unconscious transference effects (Deffenbacher et al., 2006). As a consequence, the identification task may have been quite difficult. This decision was made to avoid contamination of the CIT outcomes, which was the focus of this line of research. The results support this notion. In Experiments 2, 3, and 5, identification accuracy was somewhat lower (around 40%), compared to Experiment 1, where the identification measure was not challenged by a preceding CIT (63% accuracy on average). Furthermore, the proportion of don’t know answers, which can be taken as an indication of difficulty of the task, was higher in Experiments 2, 3, and 5 (33–36%), compared to Experiment 1 (13%). Experiment 4 constitutes an outlier in the sense that identification accuracy rates were equally high (or, if anything, even higher: 66%) and do not know responses equally low (13%) as in Experiment 1. This might be the result of our attempts to create a less complex stimulus film with only two actors and optimal viewing conditions, making the identification less difficult, compared to all other experiments.

**Table 3:** Identification accuracy rates for different roles across five experiments

Role	Identification accuracy (%)^a^	Proportion of do not know responses (%)
Experiment 1		
Thief	60.4	12.7
Victim	92.2	7.3
Bystander 1	73.5	10.9
Bystander 2	18.2	20.0
**Across roles**	62.5	12.7
Experiment 2		
Thief	44.0	29.9
Victim	34.7	32.7
Bystander 1	50.0	34.6
Bystander 2	34.7	32.7
**Across roles**	40.8	32.5
Experiment 3		
Thief	30.7	26.2
Victim	37.8	46.4
Bystander 1	35.7	33.3
Bystander 2	39.6	36.9
**Across roles**	35.6	35.7
Experiment 4		
Thief	69.2	13.3
Victim	63.1	13.3
**Across roles**	66.2	13.3
Experiment 5		
Woman 1	26.7	25.0
Woman 2	19.6	30.0
Object A	67.9	30.0
Object B	59.0	51.3
**Across roles and stimuli**	41.7	34.1

The data concerning Experiments 2–5 must be treated with caution, because the CIT task preceded the lineup task. This familiarizes participants with the stimuli presented in the subsequent lineup and possibly introduces unconscious transference effects (Deffenbacher et al., 2006). As a consequence, the identification task may have been quite difficult

^a^Calculations of identification accuracy include positive and negative identification decisions made, but exclude do not know responses (i.e., number of accurate responses divided by number of accurate + inaccurate responses)

## Discussion

It was the aim of the current line of research to test an alternative to traditional, explicit lineup identification for testing cooperative eyewitnesses’ memory for faces, using an indirect measure of face recognition. To this end, we transferred the reaction time-based CIT methodology that is well established in the field of memory detection in suspects to the field of eyewitness identification. The idea that reaction times in a CIT task should be greater for faces that were previously encountered in a stimulus event as compared to irrelevant foils was tested in a series of five experiments. The methodology of the studies sequentially progressed and addressed possible explanations for non-significant and inconsistent findings. Across 16 reaction time comparisons, seven were in favor of reaction time-based CIT predictions, whereas half of the tests returned no significant effects and one effect was opposite to our expectations. A meta-analysis showed that the overall effect size was very small. Our findings do not support the use of the reaction time-based CIT for testing cooperative eyewitnesses’ facial recognition memory. These findings contrast with the finding that the ERP-based CIT may be useful for eyewitness identification (Lefebvre et al., 2007, 2009). At least three explanations need to be considered for this apparent discrepancy.

First, it is possible that the stimulus event did not allow for sufficiently deep encoding of the faces. We think that this explanation is unlikely, because the considerable identification accuracy rates in Experiment 1—where the lineups were presented prior to the CIT—are in line with accuracy rates reported in the literature (e.g., Clark et al., 2008; Fitzgerald & Price 2015; Steblay et al., 2011) and with those reported in the previous experiments using the same stimulus film (Sagana et al., 2015, 2014, Experiments 2a–c, 3; Sauerland et al., 2012). In addition, results from a previous study deem the likelihood that the stimulus persons used in our experiments were particularly difficult to encode unlikely. Specifically, the films used in Experiments 2 and 3 served as stimulus materials in a study looking at eyewitnesses’ memory reports (Sauerland et al., 2014). Collapsed across different recall conditions, participants reported on average, about 53 person details (i.e., details referring to the appearance of the individuals shown in the film, including facial details, description of clothing, build etc.), of which, on average, 73% were accurate. Together, these findings do not seem to support the notion that it was particularly difficult to encode the actors shown in our stimulus films. Finally, while rerunning our meta-analyses including only participants who correctly identified the actor from an actor-present lineup increased the average effect size, this increase was carried by Experiment 1. It appears that viewing the lineup prior to the CIT—which was only the case in Experiment 1—improved CIT performance. Accordingly, it seems most appropriate to consider the average effect sizes excluding Experiment 1 as true effect of the reaction time-based CIT. These effect sizes were very small (including all participants from Experiments 2–5: *d* = 0.05; including only participants who accurately identified the actor from the lineup in Experiments 2–5: *d* = 0.15), regardless of accurate actor identification. This confirms our conclusion that a reaction time-based CIT does not work for lineups, even if explicit recognition occurred.

Second, a more likely explanation for our findings concerns the careful matching of the employed faces, as required by eyewitness identification procedural guidelines (e.g., Wells et al., 1998). Lineup pictures were deliberately selected to match the general description of the probes, leading to matched hair color and length, body type, and age. In fact, during debriefing, many participants spontaneously commented on the resemblance of the different stimulus faces. While the selection of individuals that match in their general description is a necessity in lineup construction, it might be obstructive for the CIT. Indeed, it was found that the more the irrelevants resemble the probe, the smaller the CIT effect (Ben-Shakhar & Gati, 1987). This may explain why Seymour and Kerlin (2008; see also Meijer et al., 2007) did find the reaction time-based CIT to be responsive to face recognition. They selected their facial stimuli from the Aberdeen Psychological Image Collection, which contains pictures of 116 people that have not been selected to match any criteria. While Lefebvre et al.’s facial stimuli (2007, 2009) were matched for some attributes, such as gender, age, race, and hair length, no information was given about other features such as hair color or hair style, and no measures of effective lineup size were provided. Thus, it is possible that the conditions for creating a fair lineup and creating an effective CIT are mutually exclusive. This notion was also confirmed by our findings referring to objects in Experiment 5. Here, the expected CIT effect was found. The fact that the crime-related objects (e.g., Hotel sign) were quite distinct from the irrelevant foils (e.g., Advent wreath, Dutch flag, Art show sign, carnival garland, occupation banner reading “This is ours”) may have contributed to the CIT effect for the objects. One way to test this idea would be by conducting a study with closely matched objects^4^ or with non-matched faces.

Third, our findings are in line with the emerging idea that different psychological processes may underlie the reaction time-based CIT and the ERP-based CIT (klein Selle et al., 2017). Lefebvre et al. (2007, 2009) provided evidence that the ERP-based CIT is sensitive to face recognition, independent of active concealment attempts. Our series of studies points to the possibility that the reaction time-based CIT critically depends on active concealment, explaining our observed null effects in cooperative witnesses. This reasoning is supported by Suchotzki et al. (2015) who suggested that the reaction times increase to probes reflects response inhibition (see also Seymour & Schumacher, 2009; Verschuere & De Houwer, 2011). Suchotzki et al. (2015) observed a reaction time-based CIT effect only when mock crime participants attempted to hide crime knowledge, but not when admitting crime knowledge. Thus, it is possible that stronger forms of active deception may be crucial for obtaining the reaction time-based CIT effect, than achieved here.^4^ This leads to the intriguing possibility that (1) CIT measures that do not depend on active deception—electrodermal responding and the P300 ERP may be effective in both cooperative and non-cooperative eyewitnesses and (2) the reaction time-based CIT may be effective in non-cooperative (i.e., deceptive) eyewitnesses (cf. Lefebvre et al., 2009).

To summarize, the results of the presented five experiments indicate that the reaction time-based CIT is not a valid means of testing facial recognition in cooperative eyewitnesses with matched faces. The findings indicate that it is important to map how stimulus distinctiveness affects the validity of the reaction time-based CIT.

## Appendix A

See Table 4.

**Table 4:** Reaction times, standard deviations, and inferential statistics for the pairwise comparisons of the reaction times for probes and irrelevant stimuli (including reaction times 150–800 ms)

Study	Role	Played by actor	*df*	*t*	*d*	*p*	Mean response time in ms (SD)	
							Probes	Irrelevants
1	Thief	A	51	− 2.78	− 0.38	.008	397 (46)	408 (52)
	Victim	B	51	3.93	0.54	< .001	445 (64)	427 (55)
	Bystander 1	C	51	− 0.90	− 0.12	.371	408 (66)	410 (57)
	Bystander 2	D	51	7.73	1.07	< .001	443 (65)	409 (54)
2	Thief	EFGH	106	− 0.36	− 0.03	.721	368 (63)	369 (53)
	Victim	EFGH	106	− 2.30	− 0.22	.023	364 (57)	371 (52)
	Bystander 1	EFGH	106	− 1.22	− 0.12	.227	364 (58)	367 (50)
	Bystander 2	EFGH	106	− 0.55	− 0.05	.582	365 (57)	366 (51)
3	Thief	EFGH	74	0.16	0.02	.870	473 (47)	473 (45)
	Victim	EFGH	74	1.81	0.21	.075	478 (50)	471 (44)
	Bystander 1	EFGH	74	0.19	0.02	.850	468 (54)	467 (45)
	Bystander 2	EFGH	74	1.14	0.13	.259	478 (60)	472 (47)
4	Thief	IJ	74	2.08	0.24	.041	467 (55)	459 (47)
	Victim	IJ	74	2.21	0.26	.030	468 (56)	460 (50)
5	Woman 1	KL	76	− 0.33	− 0.04	.746	519 (57)	520 (50)
	Woman 2	MN	76	1.71	0.19	.091	507 (51)	501 (49)
All 5 studies	Across roles				0.07 (0.02, 0.13)			
Experiments 2–5	Across roles				0.05 (− 0.00, 0.11)			

## Footnotes

1 Note that unlike common terminology in eyewitness identification studies, the target does not denote the person seen during the stimulus event. Rather, this individual is dubbed probe, whereas the term target describes the person to whom the participant has to react differently in the CIT.

2 The literature commonly refers to target-present and target-absent lineups. This terminology interferes with the CIT terminology, though, in which the probe is the person previously seen, not the target. Therefore, we refer to actor-present and actor-absent lineups in this article when referring to lineups that do or do not include the person participants saw in the stimulus event.

3 Following previous work in the field, we had initially also removed all response times slower than 800 ms to account for possible inattentive responding or strategic slowing (Kleinberg & Verschuere 2015; Noordraven & Verschuere 2013; Verschuere & Kleinberg 2015). Following the advice of reviewer Laura Visu-Petra, we reanalyzed the data with a longer deadline of 1500 ms (cf. Seymour et al. 2000). Unexpectedly this led to better results, possibly because processing time for faces can often be longer than 800 ms (Ramon et al. 2011), and has been shown to be longer than for words (e.g., Ovaysikia, Tahir, Chan, & DeSouza, 2011) which served as stimuli in previous CIT studies. We therefore report the findings of the latter analyses. To enable comparison with previous studies using the 800 ms deadline, these findings are reported in Table 4 in Appendix.

4 We thank an anonymous reviewer for bringing this point to our attention.
